# Tuberculosis Treatment Outcomes and Associated Factors among TB Patients Attending Public Hospitals in Harar Town, Eastern Ethiopia: A Five-Year Retrospective Study

**DOI:** 10.1155/2019/1503219

**Published:** 2019-04-01

**Authors:** Assefa Tola, Kirubel Minsamo Minshore, Yohanes Ayele, Abraham Nigussie Mekuria

**Affiliations:** ^1^Department of Epidemiology and Biostatistics, College of Health and Medical Sciences, Haramaya University, Harar, Ethiopia; ^2^Department of Clinical Pharmacy, College of Health and Medical Sciences, Haramaya University, Harar, Ethiopia; ^3^Department of Pharmacology, College of Health and Medical Sciences, Haramaya University, Harar, Ethiopia

## Abstract

**Introduction:**

Tuberculosis remains a major public health threat throughout the world particularly in developing countries. Evaluating the treatment outcome of tuberculosis and identifying the associated factors should be an integral part of tuberculosis treatment.

**Objectives:**

The aim of this study was to assess the treatment outcome of tuberculosis and its associated factors among TB patients in the TB clinics of Harar public hospitals, Eastern Ethiopia, 2017.

**Methods:**

A retrospective document review was conducted in two public hospitals of Harar town, located 516 km east of Addis Ababa. A systematic random sampling technique was used to select the document of TB patients who were registered in the hospitals from 1st of January, 2011, to 30th of December, 2015. The data were collected using a pretested structured data extraction format. SPSS Version 21 for window was used for data processing. Bivariate and multivariate analysis with 95% confidence interval was employed in order to infer the associations between TB treatment outcome and potential predictor variables.

**Results:**

One thousand two hundred thirty-six registered TB patients' documents were reviewed. Of these, 59.8% were male, 94.2% were urban dwellers, 97% were new cases, 61.2% were presented with pulmonary TB, and 22.8% were HIV positive. Regarding the treatment outcome, 30.4% were cured, 62.1% completed their treatment, 3.9% died, 2.4% were defaulted, and the remaining 1.2% had failed treatment. The overall rate of the treatment success among the patients was 92.5%. In the present study, being female (AOR = 1.89, 95% CI: 1.14 - 3.14), having pretreatment weight of 20 – 29 kg (AOR = 11.03, 95% CI: 1.66 - 73.35), being HIV negative (AOR = 6.50, 95% CI: 3.95 - 10.71), and being new TB patient (AOR = 3.22 95% CI: 1.10 - 9.47) were factors independently associated with successful treatment outcome. On the other hand, being in the age group 54 – 64 years (AOR =10.41, 95% CI: 1.86 - 58.30) and age greater than 65 years (AOR =24.41, 95% CI: 4.19 - 142.33) was associated with unsuccessful TB treatment outcome.

**Conclusion:**

In the current study, the rate of successful TB treatment outcome was acceptable. This rate should be maintained and further improved by designing appropriate monitoring strategies.

## 1. Introduction

Tuberculosis [1] remains a major public health threat throughout the world, developing countries shouldering much of the burden. According to the WHO report, TB is the ninth leading cause of death worldwide and it is also a leading cause of death among infectious agents, ranking above HIV/AIDS. In 2016 alone an estimated 10.4 million people fell ill with TB, of whom Africa accounted 74% cases and around 1,674,000 TB deaths were recorded globally.

According to WHO TB report, 20 countries share 85% of all estimated TB cases worldwide and Ethiopia falls under this category indicating high TB burden in the country [2]. Although the incidence of infection with TB is decreasing and there has been a positive move toward its management, more effort is needed to meet End TB Strategy, which aims at reducing the death rate by 90% by the year 2030, compared with 2015 [2, 3].

TB epidemiology is closely connected with social and economic conditions which make its prevention, care, and control more challenging [4, 5]. Many of the TB related morbidities are preventable by early diagnosis and appropriate treatment; however, owing to various reasons, its management is not always that easy [4, 5]. Evaluating the treatment outcome of TB and identifying its associated factors is an integral part of the treatment. Although the rate of the successful treatment outcome of the disease varies from country to country, 83% has been reported globally [2].

Studies conducted in various parts of Ethiopia reported different success rates of the treatment and the associated factors. For instance, a study conducted in the Northern part of the country reported 89.2% [6], but another study in the same region reported only 60.1% [7]. In the prior study, it was indicated that age greater than 40 years, family size greater than five, and being a retreatment case were strongly associated with unsuccessful treatment outcomes whereas coinfection with HIV and being male were associated with unsuccessful treatment outcomes in a later study.

Undoubtedly, the success rate of TB treatment outcome and the associated factors vary from setting to setting. Routine monitoring of the extent of the outcome and its determinants is important, but studies that have focused on TB treatment outcome and associated factors are lacking in Eastern Part of Ethiopia. Therefore, the aim of this study was to assess TB treatment outcome and factors associated among TB patients in Public Hospitals of Harar town, Eastern Ethiopia.

The objective of the study is to assess TB treatment outcome and associated factors among TB patients in Public Hospitals of Harar town, Eastern Ethiopia, 2017.

## 2. Methods and Materials

### 2.1. Study Area and Period

This study was conducted in two public hospitals (Hiwot Fana Specialized University Hospital and Jugal Hospital) in Harar town. The data were collected from April 24 to 29, 2017.

### 2.2. Study Design and Data Collection

A retrospective document review was conducted to assess the treatment outcome of TB patients and the associated factors from TB patients' documents registered from January 1, 2011, to December 31, 2015, at Public hospitals in Harar town.

### 2.3. Source Population

The documents of all the TB patients who were registered in the two public hospitals from 1st of January, 2011, to December 31st, 2015, were the source population.

### 2.4. Study Population

The documents of the randomly selected TB patients who were registered in the two public hospitals from 1st of January, 2011, to December 31st, 2015, were the study population.

### 2.5. Inclusion and Exclusion Criteria

The documents of all the registered TB patients at two public hospitals were included in this study.

However, registries in which treatment outcomes were missing and patients were transferred to other districts were excluded from the study.

### 2.6. Sample Size Determination

The sample size was determined by using double population proportions formula.

(1) $$\begin{array}{c} n=\frac{{\left(z_{\alpha /2}+z_{\beta }\right)}^{2}x2\overset{-}{P}\left(1-\overset{-}{P}\right)}{{\left(p_{1}-p_{2}\right)}^{2}} \end{array}$$

where** n**= sample size for each group

 
Z_*α*/2_= the desired level of statistical significance: 95% => 1.96
 
*Z*_*β*_ = the desired power: 80% power => 0.84
  P_1_= proportions of good/poor treatment outcome among the nonexposed
  P_2_= proportions of good/poor treatment outcome among the exposed
 
p_1_ − *p*_2_ = the difference in proportions of good/poor treatment outcome among the nonexposed and exposed
 
$\overset{-}{P}=\left(p_{1}+p_{2}\right)/2$

By using the above formula

1. cure rate among HIV negative and HIV positive TB patients

Z*α*=95%  Z*β*= 80%, r=1, P1=85. %, P2=55.7%  n=74

(2) cure rate among male and female TB patients

Z*α*=95%  Z*β*= 80%, r=2, P1=4.3% P2=8.6%  n=1026

(3) Cure rate among male and female TB patients

Z*α*=95%  Z*β*= 80%, r=2, P1=3.6 % P2=7.2 %  n=1236

 From the above options, the largest sample size (1236) was selected [8].

### 2.7. Sampling Technique

Totally 3329 TB patients registered during the review period, from January 1st, 2011, to December 31st, 2015, in two public hospitals (1535 in Jugal Hospital and 1794 in Hiwot Fana).

The sample size (1236) was distributed to each clinic using a proportionate allocation. Therefore, a total sample size for each TB clinic was

(2) Jugal  Hospital  TB  clinic=NiN×n=15353329×1236=570HiwotFana  Specialized  University  Hospital  TB  clinic=NiN×n=17943329×1236=666

From each TB clinic, registry which met the inclusion criteria was selected through a systematic sampling technique. This sampling interval (K) was obtained by dividing the total TB patients in each hospital to its sample size (k =1869/940=1.99 ~2 for Hiwot Fana Hospital and 1460/734=1.99 ~2 for Jugal Hospital). The 1st sample was taken by lottery method from 1 and 2; then the data were collected, every other registration number at both sites.

### 2.8. Data Collection

The data were collected by using a pretested structured data extraction format. The standard TB registry, laboratory findings, and monthly cohort follow-up form were reviewed to generate the required data. The standardized checklist used included all important sociodemographic data, clinical characteristics (sputum smear, type of TB, patient type, HIV status, drug regimen, and treatment outcomes), laboratory findings, and follow-up data. Data was collected by four BSc. nurses who have had training on comprehensive TB care and experience in data collection.

### 2.9. Statistical Analysis

Data were entered, cleaned, and analyzed using SPSS Version 21 for windows. Appropriate descriptive statistics such as mean (with standard deviation), median (with inter quartile range [IQR]), and frequencies (with percentages) were used to describe the study population in relation to relevant variables.

Bivariate and multivariate analysis with 95% confidence interval was employed to infer associations between the independent and dependent variables.

Binary logistic regression was used to calculate the crude odds ratio (COR) with 95% confidence interval. Each variable was entered into a logistic regression model so as to determine the presence of statistical significant association with the outcome variable. Multicollinearity among selected independent variables was checked through variance inflation factor (VIF) and none was found. All the explanatory variables with a* P* value≤ 0.2 in the bivariate analyses were included in the final multivariable logistic model in order to identify the independent predictors of TB treatment outcome. A* P* value < 0.05 was considered statistically significant. Assumption on fitness of goodness of the final model was checked by Hosmer and Lemeshow test and was found fit.

### 2.10. Ethical Statement

Ethical approval was obtained from research review technical committee of Harar health Science College. A legal authorization letter was written from Harar health Science College to study settings to get permission before data collection. The information obtained was made anonymous and deidentified prior to analysis to ensure confidentiality.

### 2.11. Operational Definitions

In this study, treatment outcomes are categorized into successful and unsuccessful treatment outcomes.

Successful treatment outcome included “cured” and “treatment completed” cases.

Unsuccessful treatment outcome included “treatment failure” cases, “defaulter,**”** and patients who “died.”

*According to the WHO, Treatment Outcomes Were Categorized into the following.*

 
*Successful outcome*: if the TB patients were cured (negative smear microscopy at the end of the treatment and on at least one previous follow-up test) or completed treatment with resolution of symptoms.
 
*Unsuccessful outcome: *if the treatment resulted in treatment failure (remaining smear positive after 5 months of treatment), patients are defaulted (patients who interrupted their treatment for two consecutive months or more after registration), or patients died.

According to the standard definitions of the National Tuberculosis and Leprosy Control Program guidelines of Ethiopia (NTLCP)[9], the following definitions were used for treatment outcome.

 
*Cured:* if the patients had finished treatment with negative bacteriological result at the end of the treatment.
 
*Treatment completed:* if the patients had finished treatment, but without bacteriological result at the end of the treatment.
 
*Treatment failure:* a patient who, while on treatment, remained smeary positive or became again smear positive at the end of the five months or later, after commencing treatment or a patient who was PTB-negative at the beginning and turned out smear positive at the end of the intensive phase.
 
*Defaulter:* a patient who had been on treatment for at least 4 weeks and whose treatment was interrupted for 8 or more consecutive weeks.
 
*Died:* if the patient died from any cause during the course of the treatment.
 
*New case:* a patient who had never had treatment for TB or had been on anti-TB treatment for less than four weeks.

Three types of TB were considered in this study. These were smear positive pulmonary TB (PTB+), smear negative pulmonary TB (PTB-), and extra-pulmonary TB (EPTB).

 
*Smear positive pulmonary TB (PTB+):* a patient who had at least 2 initial smear examinations positive for AFB by direct microscopy or one initial smear examination positive and culture positive or one initial smear positive and radiographic abnormalities consistent with TB.
 
*Smear negative pulmonary TB (PTB-):* a patient who had three initial smear examinations negative for AFB and no response to course of broad-spectrum chemotherapy and again 3 smear examinations negative by direct microscopy and radiographic abnormalities consistent with PTB and three initial smear examinations negative by direct microscopy but positive by culture and decision by a clinician to treat with anti-TB
 
*Extra-pulmonary TB (EPTB):* a patient who had TB in organs other than the lungs proven by one culture-positive specimen from extra-pulmonary site or histopathological evidence from a biopsy or TB based on strong clinical evidence with active EPTB and decision by a physician to treat with anti-TB.

## 3. Results

### 3.1. Sociodemographic Characteristics

The documents of 1236 TB patients were reviewed in this study. More than half of these TB patients (59.8%) were male, with male-to-female ratio of 1.5 to 1. Their age ranged from 1 to 95 years, with a median of 29 years (IQR: 22 - 40 years). About 363 (29.4%) of the participants were in the age range of 25 – 34 years and 355 (28.7%) were in the age range between 15 and 24 years. The weight of the TB patients ranged from 6 to 89 kg with mean (± SD) weight of 48.9 (±11.3) kg. More than half (59.7%) weighed 38 – 54 kg (Table 1).

### 3.2. Clinical Characteristics of Patients

Most of the attendants (97%) were new TB cases. Seven hundred fifty-six (61.2%) of the patients were presented with pulmonary TB of whom 400 (52.9%) were smear positive. Significant number of the patients 282 (22.8%) were found to be HIV positive of whom 250 (88.7%) and 258 (91.5%) were on ART and Cotrimoxazole Preventive Treatment (CPT), respectively.

Among smear positive PTB patients at the beginning of the treatment, 10 (2.5%), 7 (1.75%), and 14 (3.56%) were smear positive at second, fifth, and seventh months of the treatment, respectively. Most of the (97%) patients were treated with Rifampicin, Isoniazid, Pyrazinamide, and Ethambutol (RHZE) in intensive phase and 86.4% were on Rifampicin and Isoniazid (RH) during the continuation phase (Table 2).

**Figure 1:**
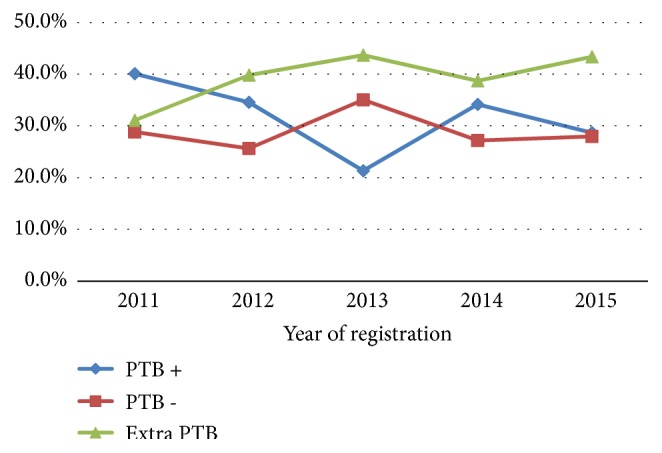
Trends of types of tuberculosis at public hospitals of Harar town, Eastern Ethiopia, 2011 - 2015.

Concerning the trend of all forms of TB, the proportion of extra-pulmonary TB slightly increased from 31.1% in 2011 to 43.3% in 2015 whereas the proportion of smear positive pulmonary TB decreased in the first three years from 40.1% in 2011 to 21.3% in 2013 (Figure 1).

### 3.3. Treatment Outcome of TB Patients on Anti-TB Therapy

Among the TB patients included in this study, 376 (30.4%) were cured, 767 (62.1%) had completed their treatment, 48 (3.9%) were dead, 30 (2.4%) were defaulted, and the remaining 15 (1.2%) had failed treatment.

The cure rate was high in the year 2011 (38.1%) and low in the year 2013 (19.8%). The default rate showed slight increment during the review period, 1.3% in the year 2011 to 3.4% in the year 2015. However, the death rate showed a reduction in the first three years (2011 – 2013) from 6.1 % to 2.0% and then started to increase in the final two years of the study period (2014 and 2015) from 3.3 % to 4.4% (Table 3).

### 3.4. Factors Associated with TB Treatment Outcome

The overall rate of the treatment success (cure and treatment complete) in this study was 92.5%. The rate of treatment success was higher among females (93.8%) than males (91.6%) and patients from rural setting (94.4%) than urban setting (92.4%). The proportion of TB patients with treatment success steadily decreased since 2012 (94.8%), 2013 (93.9%), 2014 (93.0%), and 2015 (90.8%); however, the difference was not statistically significant (*P* value =0.429). The treatment success rates were 94.3%, 90.7%, and 92.3% among the PTB+, PTB-, and EPTB patients, respectively. In the bivariate analysis, variables with* P* value of < 0.2 were sex, age, pretreatment weight, HIV status, patient category, and type of TB (Table 4).

In the multivariable logistic regression; sex, age, pretreatment weight, HIV status, and TB patient category were found to be independently and significantly associated with the treatment outcome. The chance of having successful TB treatment outcome was* 1.89 times* higher among the female TB patients (AOR =* 1.89*, 95% CI: 1.140 - 3.135) compared to the male. The HIV negative patients had 6.50 times higher odds of (AOR = 6.502, 95%CI: 3.947 - 10.712) having treatment success than the HIV positive. Similarly, the odds of treatment success were 3.22 (AOR = 3.222 95%CI: 1.096 - 9.472) times higher among new TB patients than retreatment TB cases (Table 5).

## 4. Discussion

Monitoring tuberculosis treatment outcomes and analyzing the responsible factors for unsuccessful treatment have paramount importance to evaluate the effectiveness and efficiency of TB intervention programs. In this study, we aimed to assess TB treatment outcomes and the associated factors in Harar public hospitals, Eastern Ethiopia.

In this study, the majority of the TB cases were male (59.8%). This finding was consistent with previous studies conducted in different areas of Ethiopia (Gimbi (59.3%) [10], Debre Tabor (58.1%) [11], Metema (60%) [12], Gambella (60%) [13], Dilla (61.3%) [14], Afar (62.6%) [15], Southern Ethiopia (57%) [16], Gondar (56%) [7], Hossana (55.3%) [17], Sidama Zone (55%) [18], Tigray (54.3%) [6], and Western Ethiopia (54.3%) [19]) and elsewhere in (Egypt (60.4%) [20], Ibadan Nigeria (55%) [21], South West Nigeria (59%) [22], Ebonyi state of Nigeria (57·7%) [23], and Malaysia (65%) [24]). The higher TB rate among males than the females might be due to the fact that males are more likely either to be exposed to the disease or to utilize health services than the females. Women may also underreport their disease and seek care outside health institutions because of socioeconomic constraints.

The age of the TB patients in this study ranged from 1 to 95 years, with the median age of 29 years and this is also similar to the findings of previous studies [7, 10, 12–14, 16, 18, 19]. Moreover, many of the TB cases we reviewed were between 15 and 34 years of age. This finding indicates that TB affects mainly the productive age group of the society, which can add up on the economic burden of the society particularly in developing countries.

In the present study, 3% of the patients were in the retreatment category. Similar findings were reported in studies conducted in Enfranz (2.4%) [25], Sidama Zone (5%) [18], and Metema (5.7%) [12]. However, it was less than reported from Gimbi (9.9%) [10], Western Ethiopia (8.9%) [19], Debre Tabor (10.9%) [11], Addis Ababa (11.1%) [26], and Gondar (17.5%) [7]. This could be due to the difference in time of the study, study setting, and sample size. Those studies reported the treatment outcome of TB patients before 2013 [7, 11, 19, 26] and were conducted at health centers [11, 19, 26] with smaller sample size [10, 11].

The prevalence of HIV among the patients in this study was 22.8%, which is comparable with the reports from Gambella (26%) [13], Gimbi (23.5) [10], and Metema (20.1%) [12]. However, our finding was higher than previous studies at Hosanna (16.5%) [17], Debre Tabor (12.7%) [11], Enfranz (11.7%) [25], Gondar (13.4%) [7], Iran (2.7%) [27], Dembia (11%) [28], Malaysia (6.6%) [24], Tigray region (8.6%) [6], and Western Ethiopia (17%) [19]. This high TB-HIV coinfection in our study could be due to the higher proportion of patients from urban setting where there is high prevalence of HIV.

More than one-third of the study subjects (38.8%) were presented with extra-pulmonary TB. This finding was comparable with studies from Gimbi (36%) [10], Western Ethiopia (39.7%) [19], Debre Tabor (33.3%) [11], and Addis Ababa (40.1%) [26]. Our finding was also consistent with the report of Ethiopian national population based tuberculosis prevalence survey [29]. This national TB survey stated that the mean (±SD) percentage of extra-pulmonary TB over total notifications from 2000 to 2010 was 34.8% (1.2%). This high proportion of extra-pulmonary TB in Ethiopia might be due high HIV prevalence, over diagnosis or misclassification bias. Furthermore, a similar proportion (32.4%) of TB patients in our study were presented with smear positive pulmonary TB and this is similar with the reports from Metema (30%) [12], Debre Tabor (28%) [11], Dabat (30%) [30], and Dilla (35.4%) [14].

In the current study, the overall rate of the treatment success of the TB patients was 92.5%, and this is similar to the findings reported from Tigray Region (89%) [6], Dabat (88%) [30], Debre Tabor (87.1%) [11], and Enfraz (95%) [25]. However, it is higher than national report of Ethiopia (84%) [2] and other previous studies in Ethiopia which reported treatment success ranging from 43.3% in Hossana to 85% in Dilla [7, 10, 12–19, 26]. This high treatment success rate in this study might be due to good implementation of DOTs strategy, which was indicated by lower defaulting and treatment failure rates. Another possible explanation could be also due to the exclusion of transfer out patients in this study which was categorized under unsuccessful treatment outcome group in previous studies. In addition, the majority of the TB patients in this study were urban residence; they might have had a better health seeking behavior.

TB associated death rate in present study was 3.9%. This figure was in line with report from Dilla (3.4%) [14], Addis Ababa (3.7%) [26], Afar (4.5%)[15], Enfranz (3.4%) [25], Gambella (3.7%), Dembia (3.3%), Metema (3%), Sidama zone (3.1%), Gimbi (3.7%), Tigray Region (3.9%) [6], Dabat (3.1%) [30], and Hossana (2.9%) [17]. However, some studies reported higher proportion of TB associated death rate, ranging from 5.6% in Debre Tabor and Egypt to 17.7% in Gondar [16, 19, 22, 24, 27, 31].

Defaulting from Anti-TB treatment clinic is one of the main challenges of TB controlling program. The defaulting rate of TB patients from their treatment in this study was 2.4%, which is in line with the finding in Debre Tabor. However, the majority of previous studies reported that defaulting from TB treatment ranged from 2.8% in Dabat to 30.8% in South West Nigeria [6, 7, 10, 12–16, 18–22, 24, 26, 28, 32]. The lower defaulting rate in our study could be due to better implementation of DOT strategies, including defaulter tracing system, supervision, and health education activities.

The other main problem of TB controlling program is treatment failure. In our study, 1.2% of the TB patients were identified as treatment failure. This figure is lower than the reports from Ibadan Nigeria (8.1%) [21], Egypt (6.25%), Niger delta (4.8%), South West Nigeria (3.8%), Tigray Region (3.7%), and Debre Tabor (3.5%), but it is higher than finding from many studies conducted in different parts of Ethiopia [7, 12–16, 18, 19, 25, 26, 30] which ranged between 0.2% and 0.8%.

Identification of factors associated with treatment outcome of TB patients is very crucial in order to tackle those factors leading to poor treatment outcome. The present study revealed that being female; age greater than 55 years, having pretreatment weight of 20 – 29 kg and 38 – 54 kg, being HIV negative, and being new TB patient were independently significantly associated with successful treatment outcome.

As reported by many studies [12, 15, 21, 23, 24, 27, 33], females have had bigger chance of successful TB treatment outcome than males. However, the odds of successful treatment outcome were better for males compared to females in studies conducted in Debre Tabor [11], Southern Region [16], and Tigray Region [6]. This difference might have been related to other variables like severity of the disease, timing of the treatment, adherence to the treatment, and behavioral and socioeconomic characteristics. Since almost all of these studies were based on retrospective document review, they did not take into consideration the effect of those important variables.

The HIV status of the TB patients was one of the factors that were associated with the treatment outcome. Not surprisingly, in this study, the HIV negative patients had bigger chance of successful TB treatment than the HIV positive ones. This finding is consistent with several previous studies [7, 10, 12, 19, 22, 24, 27, 28].

## 5. Conclusion and Recommendation

The treatment outcome of the TB patients who received TB treatment at the study area was satisfactory and in line with the WHO target. The treatment outcome was significantly associated with gender, age, pretreatment weight, HIV status, and TB category. Moreover, it was not significantly affected by place of residence, year of treatment, and types of TB.

Based on the findings of this study, we recommend that frequent supportive supervision and health education programs for patients with a high risk of unsuccessful treatment outcome should be carried out. In addition, further prospective studies are needed to identify other potential sociodemographic and behavioral factors that could affect the treatment outcomes of TB patients.

## Figures and Tables

**Table 1:** Socio-demographic characteristics of TB patients attending public hospitals of Harar town, Eastern Ethiopia, 2017 (n=1236).

*Variables*	*Frequency*	*Percent*
*Sex *		
Male	739	59.8
Female	497	40.2
*Age *		
1 - 14 years	68	5.5
15 - 24 years	355	28.7
25 - 34 years	363	29.4
35 - 44 years	205	16.6
45 - 54 years	119	9.6
55 - 64 years	72	5.8
> 64 years	54	4.4
*Place of residence *		
Urban	1164	94.2
Rural	72	5.8
*Pretreatment Weight *		
Less than 20 Kg	22	1.8
20 - 29 Kg	30	2.4
30 - 37 Kg	85	6.9
38 - 54 Kg	738	59.7
Greater than 54 Kg	361	29.2
*Year of treatment *		
2011	312	25.2
2012	191	15.5
2013	197	15.9
2014	243	19.7
2015	293	23.7

**Table 2:** Clinical characteristics of TB patients attending public hospitals of Harar town, Eastern Ethiopia, 2017 (n=1236).

*Variables*	*Frequency*	*Percent*
*TB clinic*		
Hiwot Fana SUH	666	53.9
Jugal Hospital	570	46.1
*Category of patient*		
New case	1199	97.0
Re treatment	37	3.0
*Types of TB*		
Pulmonary TB	756	61.2
Extra PTB	480	38.8
*Smear result (n=756)*		
Smear positive	400	52.9
Smear negative	356	47.1
*HIV status *		
HIV positive	282	22.8
HIV negative	954	77.2
*ART initiated (n=282) *		
Yes	250	88.7
No	32	11.3
*CPT initiated (n=282)*		
Yes	258	91.5
No	24	8.5
*Smear result after 2 months (n=400)*		
Positive	10	2.5
Negative	390	97.5
*Smear result after 5 month (n= 400)*		
Positive	7	1.75
Negative	393	98.25
*Smear result after 7 months (n=387)*		
Positive	9	2.3
Negative	378	97.7
*Anti-TB Drug on Intensive phase *		
RHZE	1199	97.0
SERHZ	37	3.0
*Anti-TB Drug on continuation phase*		
RH	1068	86.4
EH	168	13.6

**Table 3:** Distribution of treatment outcome with socio-demographic and clinical characteristics of TB patients attending public hospitals of Harar town, Eastern Ethiopia, 2017 (n=1236).

*Characteristics *	*Treatment outcome*					*Total*
	*Cure*	*Completed*	*Died*	*Failure*	*Defaulted*	
*Year of registration*						
2011	119 (38.1%)	166(53.2%)	19(6.1%)	4(1.3%)	4(1.3%)	*312*
2012	62(32.5%)	119(62.3%)	4(2.1%)	3(1.6%)	3(1.6%)	*191*
2013	39(19.8%)	146(74.1%)	4(2.0%)	3(1.5%)	5(2.5%)	*197*
2014	78(32.1%)	148(60.9%)	8(3.3%)	1(0.4%)	8(3.3%)	*243*
2015	78(26.6%)	188(64.2%)	13(4.4%)	4(1.4%)	10(3.4%)	*293*
*Place of residence *						
Urban	359 (30.8%)	716 (61.5%)	48 (4.1%)	14 (1.2%)	27 (2.3%)	*1164*
Rural	17 (23.6%)	51 (70.8%)	0 (0.0%)	1 (1.4%)	3 (4.2%)	*72*
*Age group*						
1 - 14 years	12 (17.6%)	50 (73.5%)	4 (5.9%)	1 (1.5%)	1 (1.5%)	*68*
15 - 24 years	129 (36.3%)	213 (60.0%)	8 (2.3%)	1 (0.3%)	4 (1.1%)	*355*
25 - 34 years	120 (33.1%)	219 (60.3%)	10 (2.8%)	4 (1.1%)	10 (2.8%)	*363*
35 - 44 years	60 (29.3%)	131 (63.9%)	6 (2.9%)	2 (1.0%)	6 (2.9%)	*205*
45 - 54 years	32 (26.9%)	75 (63.0%)	2 (1.7%)	5 (4.2%)	5 (4.2%)	*119*
55 - 64 years	15 (20.8%)	46 (63.9%)	7 (9.7%)	2 (2.8%)	2 (2.8%)	*72*
>64 years	8 (14.8%)	33 (61.1%)	11 (20.4%)	0 (0.0%)	2 (3.7%)	*54*
*Sex *						
Male	241 (32.6%)	436 (59.0%)	27 (3.7%)	11 (1.5%)	24 (3.2%)	*739*
Female	135 (27.2%)	331 (66.6%)	21 (4.2%)	4 (0.8%)	6 (1.2%)	*497*
*Pre-treatment weight *						
< 20 Kg	2 (9.1%)	16 (72.7%)	2 (9.1%)	1 (4.5%)	1 (4.5%)	*22*
20 - 29 Kg	3 (10.0%)	24 (80.0%)	2 (6.7%)	1 (3.3%)	0 (0.0%)	*30*
30 - 37 Kg	28 (32.9%)	47 (55.3%)	5 (5.9%)	0 (0.0%)	5 (5.9%)	*85*
38 - 54 Kg	232 (31.4%)	456 (61.8%)	29 (3.9%)	8 (1.1%)	13 (1.8%)	*738*
>55 Kg	111 (30.7%)	224 (62.0%)	10 (2.8%)	5 (1.4%)	11 (3.0%)	*361*
*HIV status *						
HIV positive	73 (25.9%)	159(56.4%)	25(8.9%)	9(3.2%)	16(5.7%)	*282*
HIV negative	303(31.8%)	608(63.7%)	23(2.4%)	6(0.6%)	14(1.5%)	*954*
*Type of TB *						
Pulmonary TB	376 (49.7%)	325 (43.0%)	25 (3.3%)	15 (2.0%)	15 (2.0%)	*756*
Extra PTB	0 (0.0%)	442 (92.1%)	23 (4.8%)	0 (0.0%)	15 (3.1%)	*480*
*Patient category *						
New cases	352 (29.4%)	759 (63.3%)	46 (3.8%)	13 (1.1%)	29 (2.4%)	*1199*
Re treatment	24 (64.9%)	8 (21.6%)	2 (5.4%)	2 (5.4%)	1 (2.7%)	*37*
*Total *	*376 (30.4%)*	*767 (62.1%)*	*48 (3.9%)*	*15 (1.2%)*	*30 (2.4%)*	*1236*

**Table 4:** Bivariate analysis of treatment outcome with socio-demographic and clinical characteristics of TB patients attending public hospitals of Harar town, Eastern Ethiopia, 2017 (n=1236).

*Characteristics *	*Treatment outcome*			
	Successful Treatment	Unsuccessful treatment	*P. value*	*COR (95%CI)*
*Place of residence *				
Urban	1075 (92.4%)	89(7.6%)	0.516	1.00
Rural	68 (94.4%)	4(5.6%)		1.407 (0.502 - 3.947)
*Sex *				
Male	677 (91.6%)	62(8.4%)	0.161	1.00
Female	466(93.8%)	31 (6.2%)		1.377 (0.880 - 2.153)
*Age group*				
1 - 14 years	62 (91.2%)	69 (8.8%)	*≤ 0.001*	1.00
15 - 24 years	342(96.3%)	13(3.7%)	0.068	0.393 (0.144 - 1.072)
25 - 34 years	339(93.4%)	24(6.6%)	0.512	0.732 (0.287 - 1.863)
35 - 44 years	191(93.2%)	14(6.8%)	0.585	0.757 (0.279 - 2.055)
45 - 54 years	107(89.9%)	12(10.1%)	0.779	1.159 (0.414 - 3.242)
55 - 64 years	61(84.7%)	11(15.3%)	0.248	1.863 (0.648 - 5.355)
>64 years	41(75.9%)	13(24.1%)	*0.026*	*3.276 (1.153 - 9.313)*
*Pre-treatment weight*				
< 20 Kg	18 (81.8%)	4(18.2%)	0.177	1.00
20 - 29 Kg	27(90.0%)	3(10.0%)	0.074	2.863 (0.902 - 9.084)
30 - 37 Kg	75 (88.2%)	10(11.8%)	0.576	1.432 (0.407 - 5.036)
38 - 54 Kg	688(93.2%)	50(6.8%)	0.169	1.718 (0.795 - 3.714)
>55 Kg	335 (92.8%)	26(7.2%)	0.793	0.936 (0.573 - 1.531)
*Year of registration*				
2011	285(91.3%)	27 (8.7%)	0.429	1.00
2012	181(94.8%)	10(5.2%)	0.809	0.933 (0.534 - 1.632)
2013	185(93.9%)	12 (6.1%)	0.112	0.544 (0.257 - 1.152)
2014	226 (93.0%)	17 (7.0%)	0.213	0.639 (0.316 - 1.294)
2015	266 (90.8%)	27(9.2%)	0.353	0.741 (0.394 - 1.395)
*HIV status *				
HIV positive	232 (82.3%)	50(17.7%)	*≤ 0.001*	1.00
HIV negative	911(95.5%)	43(4.5%)		*4.566 (2.963 - 7.036)*
*Patient category *				
New cases	1111 (92.7%)	88 (7.3%)	0.169	1.00
Re treatment	32 (86.5%)	5(13.5%)		0.507 (0.193 - 1.333)
*Type of TB *				
PTB +	377 (94.3%)	23 (5.8%)	0.188	1.00
PTB -	323 (90.7%)	33 (9.3%)	0.253	0.730 (0.426 - 1.251)
Extra PTB	443 (92.3%)	37 (7.7%)	0.421	1.223(0.749 - 1.998)

**Table 5:** Multivariate analysis of treatment outcome with socio-demographic and clinical characteristics of TB patients attending public hospitals of Harar town, Eastern Ethiopia, 2017 (n=1236).

*Characteristics *	*Treatment outcome*			
	Successful Treatment	Unsuccessful treatment	*P. value*	*AOR (95%CI)*
*Sex *				
Male	677 (91.6%)	62(8.4%)	0.015	1.00
Female	466(93.8%)	31 (6.2%)		*1.890 (1.140 - 3.135)*
*Age group*				
1 - 14 years	62 (91.2%)	69 (8.8%)	*≤ 0.001*	1.00
15 - 24 years	342(96.3%)	13(3.7%)	0.367	2.138 (0.411 - 11.126)
25 - 34 years	339(93.4%)	24(6.6%)	0.217	2.617 (0.545 - 14.561)
35 - 44 years	191(93.2%)	14(6.8%)	0.318	2.386 (0.436 - 13.009)
45 - 54 years	107(89.9%)	12(10.1%)	0.098	4.243 (0.766 - 23.520)
55 - 64 years	61(84.7%)	11(15.3%)	0.008	*10.413 (1.860 - 58.298)*
>64 years	41(75.9%)	13(24.1%)	*≤ 0.001*	*24.41 (4.188 - 142.331)*
*Pre-treatment weight*				
< 20 Kg	18 (81.8%)	4(18.2%)	0.035	1.00
20 - 29 Kg	27(90.0%)	3(10.0%)	0.012	*11.027(1.658 - 73.347)*
30 - 37 Kg	75 (88.2%)	10(11.8%)	0.088	4.053 (0.813 - 20.192)
38 - 54 Kg	688(93.2%)	50(6.8%)	0.022	*2.824 (1.167 - 6.831)*
>55 Kg	335 (92.8%)	26(7.2%)	0.612	1.151 (0.669 - 1.978)
*HIV status *				
HIV positive	232 (82.3%)	50(17.7%)	≤ 0.001	1.00
HIV negative	911(95.5%)	43(4.5%)		*6.502 (3.947 - 10.712)*
*Patient category *				
Re treatment	32 (86.5%)	5(13.5%)	0.033	1.00
New cases	1111 (92.7%)	88 (7.3%)		*3.222 (1.096 - 9.472)*
*Type of TB *				
PTB +	377 (94.3%)	23 (5.8%)	0.415	1.00
PTB -	323 (90.7%)	33 (9.3%)	0.208	0.686 (0.382 - 1.233)
Extra PTB	443 (92.3%)	37 (7.7%)	0.876	0.959 (0.566 - 1.625)

## Data Availability

The data used to support the findings of this study are available from the corresponding author upon request.
